# Leprosy in urban space, areas of risk for disability and worsening of this health condition in Foz Do Iguaçu, the border region between Brazil, Paraguay and Argentina

**DOI:** 10.1186/s12889-020-8236-5

**Published:** 2020-01-29

**Authors:** Ivaneliza Simionato de Assis, Thais Zamboni Berra, Luana Seles Alves, Antônio Carlos Viera Ramos, Luiz Henrique Arroyo, Danielle Talita dos Santos, Marcos Augusto Moraes Arcoverde, Josilene Dália Alves, Juliane de Almeida Crispim, Flávia Meneguetti Pieri, Marco Andrey Cipriani Frade, Ione Carvalho Pinto, Carla Nunes, Ricardo Alexandre Arcêncio

**Affiliations:** 1 Nursing College of Ribeirão Preto, University of São Paulo, Ribeirão Preto, São Paulo, Brazil; 2 University Center Dinâmica of Cataratas, Foz do Iguaçu, Paraná, Brazil; 3 State University of West Paraná-UNIOESTE, Foz do Iguaçu, Paraná, Brazil; 4 Federal Institute of Mato Grosso, Barra do Garças, Mato Grosso, Brazil; 5 State University of Londrina, Londrina, Paraná, Brazil; 6 Medical School, University of São Paulo, Ribeirão Preto, São Paulo, Brazil; 7 Escola Nacional de Saúde Pública, Universidade Nova de Lisboa, Lisbon, Portugal

**Keywords:** Leprosy, Spatial analysis, Border region

## Abstract

**Background:**

Leprosy is a public health problem and a challenge for endemic countries, especially in their border regions where there are intense migration flows. The study aimed to analyse the dynamics of leprosy, in order to identify areas of risk for the occurrence of the disease and disability and places where this health condition is worsening.

**Method:**

This ecological study considered the new cases of leprosy reported in the municipality of Foz do Iguaçu from 2003 to 2015. Spatial and spatial-temporal scan statistics were used to identify the risk areas for the occurrence of leprosy, as well as the Getis-Ord Gi and Getis-Ord Gi* methods. Areas of risk for disabilities were identified by the scan statistic and kernel density estimation.

**Results:**

A total of 840 cases were reported, of which 179 (21.3%) presented Grade 1 or 2 disabilities at the time of diagnosis. Leprosy risk areas were concentrated in the Southern, Eastern and Northeastern Health Districts of the municipality. The cases of Grade 2 disability were observed with higher intensity in regions characterized by high population density and poverty.

**Conclusion:**

The results of the study have revealed changes in the pattern of areas at risk of leprosy according to the investigated periods. In addition, it was possible to verify disabilities as a condition present in the investigated cases, or that may be related to the late diagnosis of the disease. In the areas of risk identified, patients have reported worse physical disability after diagnostic confirmation, or indicate inadequate clinical examination, reinforcing the need for structuring leprosy control services in a qualified manner.

## Introduction

Leprosy is a chronic, infectious disease with a high potential for disability, a problem of transcendence and a significant impact, especially in developing countries [1, 2]. In 2017, India, Brazil and Indonesia accounted for 80.2% of the cases reported worldwide. In the Americas, Brazil accounted for 92.3% of cases, and was included on the list of 22 priority countries for leprosy control actions [3].

The prevention of physical disabilities is a concern regarding the management of leprosy, since these can cause physical limitations and directly affect the patient’s quality of life [4]. Grade 2 disability (G2D) has progressively increased, jumping from 1736 new cases in 2016 to 1949 cases in 2017, which may cause a delay in the diagnosis of the disease [3]. Despite the advances in the treatment of leprosy that have occurred over the previous decades and the implementation of the Brazilian National Health System (*Sistema Universal de Saúde* - SUS) in 1988, leprosy remains a serious public health problem, and many states, mainly in the North, Northeast and Central-West [4], did not achieve the goals proposed by the World Health Organisation (WHO) for the periods 2006–2010 and 2011–2015 [5, 6]. The goals were to reduce the leprosy burden and provide affected communities with access to quality leprosy control services, considering the principles of equity and social justice, as well as reducing the rate of new cases with G2D [5, 6]. The global policies launched include the goal of eliminating leprosy (< 1 case per 100,000 inhabitants), reducing G2D cases and eradicating new cases in children [3].

Studies show that the housing conditions, environment, characteristics of areas such as the absence/presence of social facilities and social protection policies and the inadequate organisation of health services are factors which are all associated with the transmission of disease and the development of leprosy disabilities [7–10]. Different techniques have been used to highlight the relationship between leprosy and the space/area, with studies having been carried out that estimate the risk of community exposure through the spatial scan statistic [10–14] and the density of cases by means of the kernel density estimation [15–17]. Despite the exhaustive use of these techniques, the statistics Getis-Ord Gi and Getis-Ord Gi* appear to present greater sensitivity for the detection of risk areas [18]. Leprosy is not a static phenomenon, possibly oscillating between regions depending on the gradient of the exposure factor that determines it. The areas or territories are in a constant process of change [19–21] and, in turn, so are the factors associated with leprosy, therefore the Gi and Gi* statistics can be very useful, since they are able to capture this dynamicity [22, 23].

An important knowledge gap is that, of the studies that have analysed the dynamics of leprosy, few have aimed to understand how physical disabilities are distributed within the area [18]. In addition, it is important to highlight the areas where the disabilities of the patients are worsening, since these results may contribute to the qualification of services in the areas where these patients are registered. Thus, to advance knowledge, specifically in a Brazilian border territory with the high mobility of people, the study aimed to analyse the dynamics of leprosy, verifying whether the leprosy risk areas change over time, highlighting the areas where the disabilities are and identifying the regions in which patients are experiencing a worsening grade of disability.

## Methods

### Ecological study [24]

#### Study setting

**Fig. 1:**
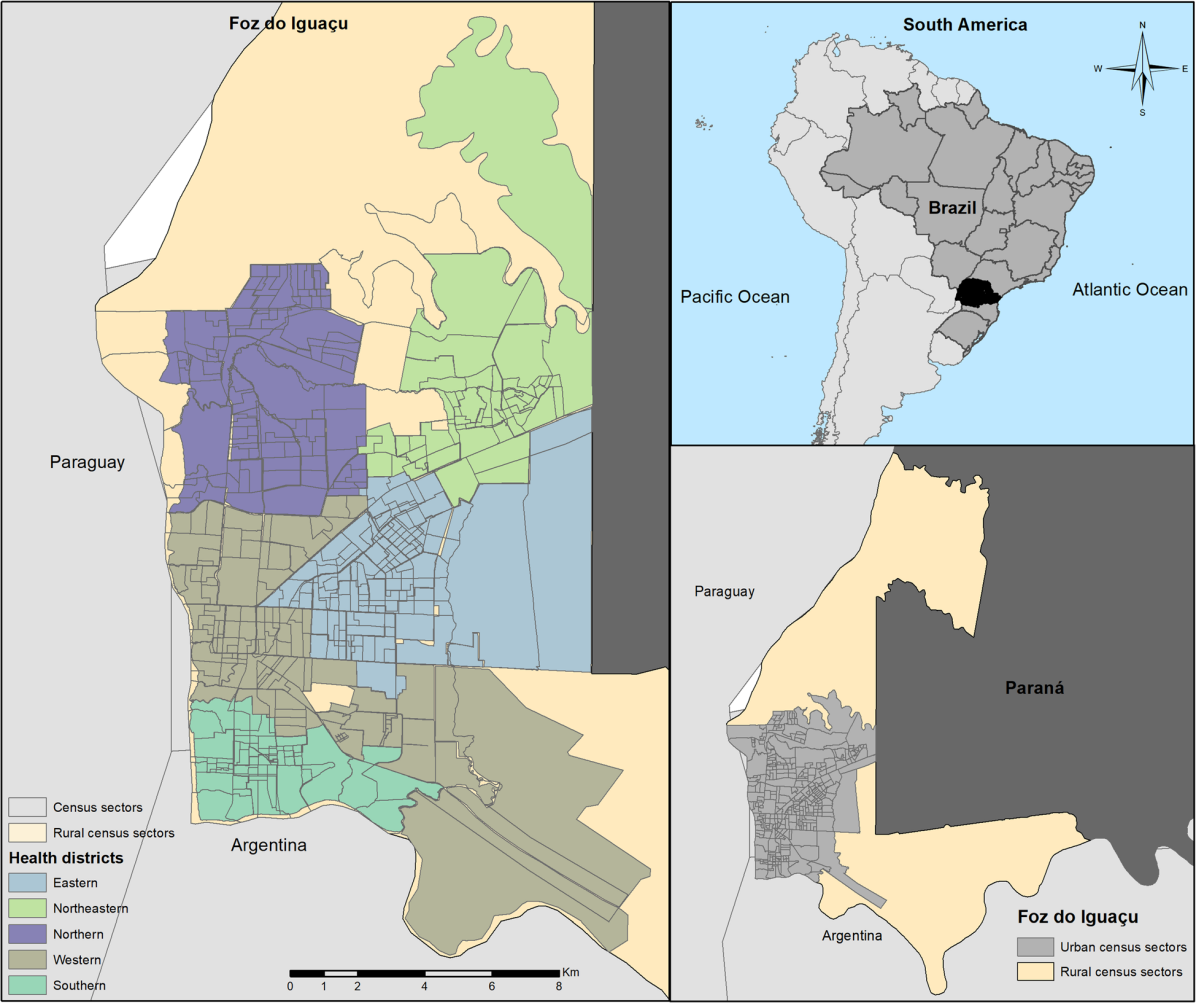
Study Setting. Brazil, Paraguay and Argentina Border Region. Source: Created by the main author

The municipality of Foz do Iguaçu has a total area of 618,353 km^2^, organised in 327 census sectors, of which 320 are urban (Fig 1). The estimated population of the municipality is 258,823 inhabitants [25]. Regarding socioeconomic conditions, it presents a Human Development Index (HDI) of 0.751, a Gini Index 0.545 and a subjective poverty index of 25.00%. The mean salary of formal workers is 2.7 minimum wages, the unemployment rate is 7.05 and 25.50% of the population has an income of less than ½ of the minimum wage [26].

Regarding the health services, the municipality has 403 health facilities, including 30 Primary Health Units (PHUs) [27], two Health Surveillance Units, two Emergency Care Units (ECUs) and four hospitals [28]. The municipality is composed of five Health Districts: Northern, Southern, Eastern, Northeastern and Western (central), all under the management of the Municipal Health Department of Foz do Iguaçu, Paraná [29]. The percentage of the population with Primary Health Care (PHC) coverage in the municipality is 36.39% (96,000 inhabitants) [30].

#### Unit of study observation and criteria

The census sectors of the urban area of the study setting were defined as the unit of analysis.

#### Study population

Cases of leprosy (ICD 10 from A30.0 to A30.9) reported between the years 2003 and 2015 and recorded in the Notifiable Disease Information System (SINAN).

#### Study variables

For the investigation, the variables age, sex, address, year of diagnosis and grade of disability at the time of diagnosis (grade 0, 1 or 2), were selected.

#### Data collection

Data were obtained from the Health Surveillance Department of the Health Department of Foz do Iguaçu, Paraná, Brazil. Data from the 2010 Demographic Census were collected through the website of the Brazilian Institute of Geography and Statistics (IBGE), referring to the census sectors.

#### Data analysis

The incidence was calculated by standardizing it by age and sex.

#### Georeferencing of the cases

For the spatial analysis, georeferencing of the cases was used to obtain the latitude and longitude from the address of the residence, through the free access Google Earth Pro software. The geocoding of the addresses was performed using the TerraView 4.2.2 software.

#### Spatial and spatial-temporal scan statistic

To analyse the dynamics of leprosy in the triple-border area over the years, it was decided to group the data into three distinct periods according to the mean rate of detection of new cases: 2003–2006, 2007–2011 and 2012–2015.

The spatial and spatial-temporal scan statistic was used to search for leprosy risk areas [31]. The principle of this technique is based on Poisson’s discrete model, using age and sex as covariables, and adopting the following parameters: the non-overlapping of agglomerates, clusters with a circular form and 999 replications. The size of the exposed population was determined from the Gini coefficient, as this is a more intuitive and systematic way to determine the best risk cluster formation [32]. For this stage, the SatScan® version 9.5 software was used. The Relative Risk (RR) for each area was obtained, and the values resulting from this calculation were considered to be high-risk for leprosy when the RR of the cluster was greater than one (RR > 1) and low-risk when it was smaller than one (RR < 1) [33]. For each RR, the 95% confidence interval (95%CI) was estimated.

The scan statistic was also initially applied, using cases with Grade 1 disability (G1D) and G2D together. The Relative Risk (RR) and the respective 95% confidence intervals were estimated.

#### Getis-Ord general G and Getis-Ord Gi*

For the comparison of the results previously found in the spatial scan statistic and the confirmation of spatial dependence, the Getis-Ord General G and Getis-Ord Gi* techniques were used.

The primary objective of the Gi and Gi* statistics is to verify the extent to which a location is surrounded by a cluster of high or low values for the variable under study, presenting a level of statistical significance for each identified risk area; this is because, in the case of high values, the smaller the type I error (alpha value), the greater the accuracy of a cluster or hotspot existing in that region [34]. The application of this technique is justified by the distance of the spatial groupings, which become more pronounced, requiring the application of Incremental Spatial Autocorrelation (ISA) [35, 36]. In this step of the study, 30 distances were applied to verify the most adequate one for study, finding 3283 m to be the best, with a z-score of 4.47 (*p* = 0.001). These analyses were conducted using the ArcGis 10.5 software. It should be highlighted that the Getis-Ord General G technique is based on the Global Moran’s Index, which has the purpose of evaluating the spatial association of an attribute based on statistical distances and determines the degree of clustering for high and low values [37].

The High/Low Clustering (Getis-Ord General G) tool of the ArcGis 10.5 software is based on inferential statistics, which means that the results of the analysis are interpreted within the context of the null hypothesis. In this case, the null hypothesis means that there is no spatial grouping [38]. However, if the *p*-value is significant, the null hypothesis is rejected and the *z*-score value becomes important, since it reflects statistical significance, where +/− 3 has a confidence level of 99% [39, 40]. If the value of the *z*-score is positive, the observed *G*-index is higher than expected, indicating that high values for the attribute are grouped in the study area. When the value of the *z*-score is negative, the observed *G*-index is lower than the index expected, indicating that low values are grouped in the study area [38].

Next, in order to explore the spatial patterns in more detail and to verify the leprosy dynamics, the Getis-Ord Gi* technique was applied, which consists of a local association index, where the values for each location, of each census sector, are considered from a neighbourhood matrix. In Getis-Ord Gi*, a *z*-score is generated; for sectors that are statistically significant, the *z*-score will be positive, with higher *z*-scores equating to more intense clustering of high values (Hotspots). For negative z-scores, however, lower z-scores equate to more intense low value groupings (Coldspots). In addition to the *z*-score, the *p*-value and level of significance (Gi-Bin) is provided. The *z*-score and *p*-value are measures of statistical significance which indicate whether the null hypothesis is rejected or not; these values do not reflect any type of “False Discovery Rate” (FDR) correction [38]. The Gi-Bin values identify statistically significant hotspots and coldspots, regardless of whether the FDR correction is being applied. Values of +/− 3 reflect statistical significance with a 99% confidence level, +/− 2 a 95% confidence level, and +/− 1 a 90% confidence level, with zero not being statistically significant [38]. With regard to the aim of identifying those areas with diagnoses of physical disabilities due to leprosy, the cases that had the disability information at the time of diagnosis were considered.

#### Kernel density estimation

Next, considering the number of cases with G2D (an indicator that is more sensitive to service quality and therefore late diagnosis), the Kernel Density Estimation technique was applied. This technique was used because it provides an overview of the distribution of sample points, which generates a density surface and identifies the so-called “hot areas” and may be indicative of cluster formation and possible spatial dependence [41]. The bandwidth (radius) of 3283 m was adopted, provided by the ISA tool of the ArcGis 10.5 software [38].

It is noteworthy that data about the grades of disability were gathered through the SINAN system, where it is possible to identify the grade of disability at the time of diagnosis and also data regarding the follow-up of the case, including the degree of disability when a cure is confirmed. In this case, the authors assessed those cases in which there was an evolution (worsening) of the grade of disability, comparing the degree at the moment of diagnosis with that in the cure phase.

From the statistics obtained with the application of Getis-Ord General G, Getis-Ord Gi*, Spatial/spatial-temporal scan statistics and Kernel Density Estimation, thematic maps were produced using the ArcGIS 10.5 software.

In compliance with Resolution 499/2012 of the National Health Council, the study was approved by the Research Ethics Committee of EERP/USP under the Certificate of Ethical Appraisal (CAAE) No. 59299816.2.0000.5393 issued on September 10, 2016.

## Results

A total of 840 cases of leprosy were reported, representing an annual detection rate of 23.2 cases per 100,000 inhabitants. Regarding the physical disabilities of these cases, 54 (6.4%) presented G2D at the time of diagnosis, 125 (14.9%) G1D, 466 (55.5%) presented no physical disability and 195 (23.2%) had not been evaluated at the time of diagnosis.

### Scan statistic, Getis-Ord general G and Getis-Ord Gi*

**Fig. 2:**
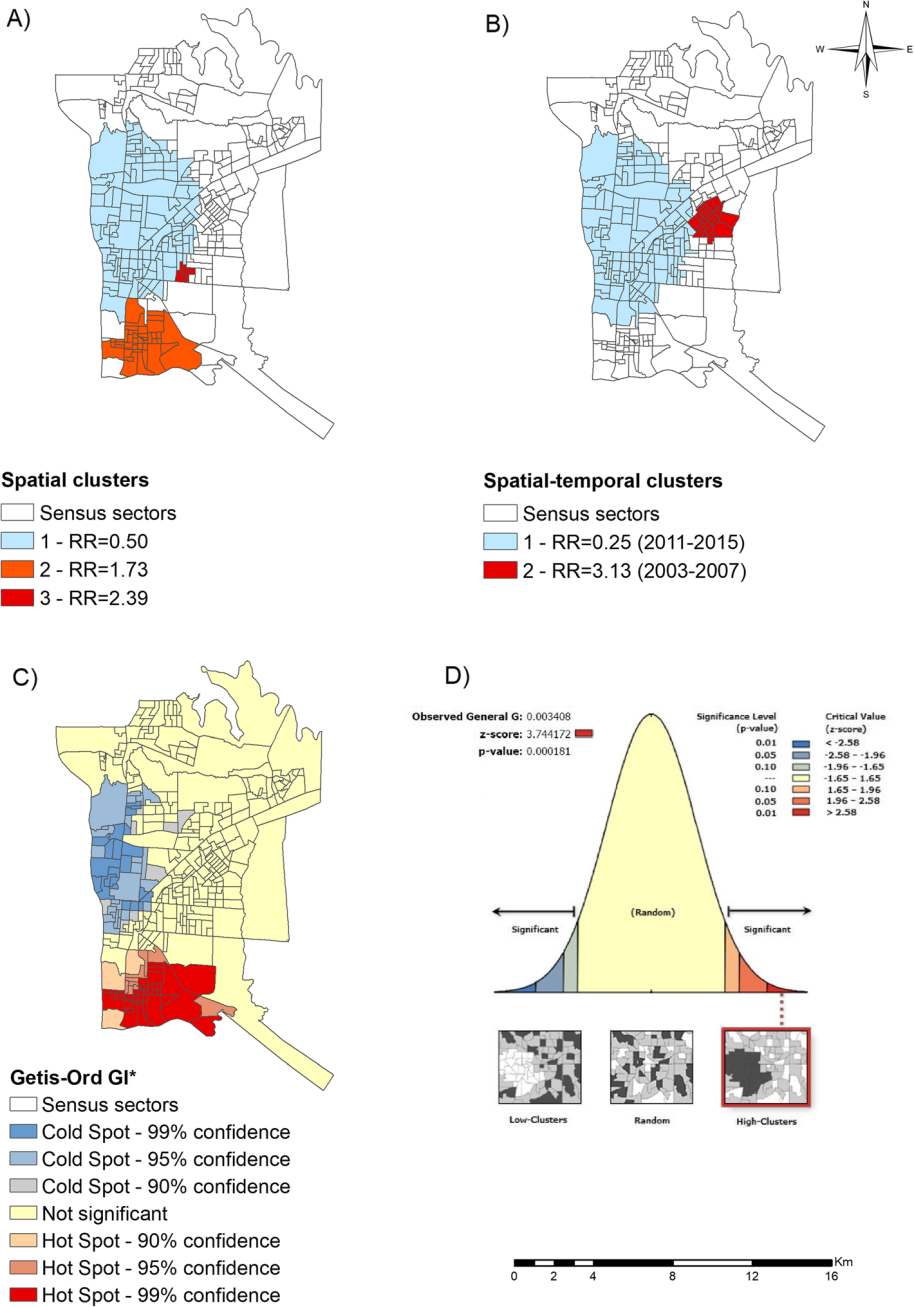
Leprosy risk areas of a municipality in the Brazil, Paraguay and Argentina border region (2003–2015). **a** Spatial clusters of new leprosy cases, **b** Spatial-temporal clusters of new leprosy cases, **c** Leprosy risk Hotspots and Coldspots according to the Gi* statistics **d** Level of statistical significance of the Getis-Ord Gi*. Source: Created by the main author

With the application of the spatial scan statistic, the formation of three clusters was observed (Fig. 2a). According to Fig. 2a, cluster 2, considered at risk of leprosy, was comprised of 40 census sectors and located in the Southern Health District, with a population of 28,447 inhabitants and an annual detection rate of 37 new cases per 100,000 inhabitants and an RR of 1.73 (95%CI = 1.40–2.07, *p* = 0.001). Cluster 3 was located in the Eastern District of the municipality, including 6 census sectors, a population of 4996 inhabitants, a detection rate of 53.9 new cases per 100,000 inhabitants and an RR of 2.39 (95%CI = 1.69–3.34, *p* = 0.031). A low-risk cluster (cluster 1) was identified in the Western District, which included 127 census sectors, a population of 91,387 inhabitants, detection rate of 14.2 new cases per 100,000 inhabitants and an RR of 0.50 (95%CI = 0.4–0.6, *p* = 0.001).

In the spatial-temporal scan statistical analysis of leprosy cases, three clusters were identified (Fig. 2b). Cluster 1 was low-risk, comprising the period from 2011 to 2015, covering 136 census sectors of the Western District and part of the Northern District, with a population of 96,734 inhabitants, a detection rate of 6.4 new cases per 100,000 inhabitants and an RR of 0.25 (95%CI = 0.17–0.36, *p* = 0.001). Cluster 2, considered to be a risk cluster, covers the period 2003–2007, with 24 census sectors in the Eastern District and a population of 18,426 inhabitants, a detection rate of 68.4 new cases per 100,000 inhabitants and an RR of 3.13 (95%CI = 2.42–4.05, *p* = 0.001).

Fig. 2c demonstrates the local spatial association (Getis-Ord Gi*), which made it possible to identify hotspots and coldspots for the risk of leprosy. Hotspots were observed in the Southern District of the municipality, with a 99 and 95% confidence level and part of the Western District, with a 90% confidence level, with a *z*-score of 3.74. Coldspots were identified in the Western District and part of the Northern District, ranging from 90 to 99% confidence level. Using the pseudo-significance test, it was possible to confirm the non-randomness of the clusters, with this situation being statistically significant (*p* < 0.01).

**Fig. 3:**
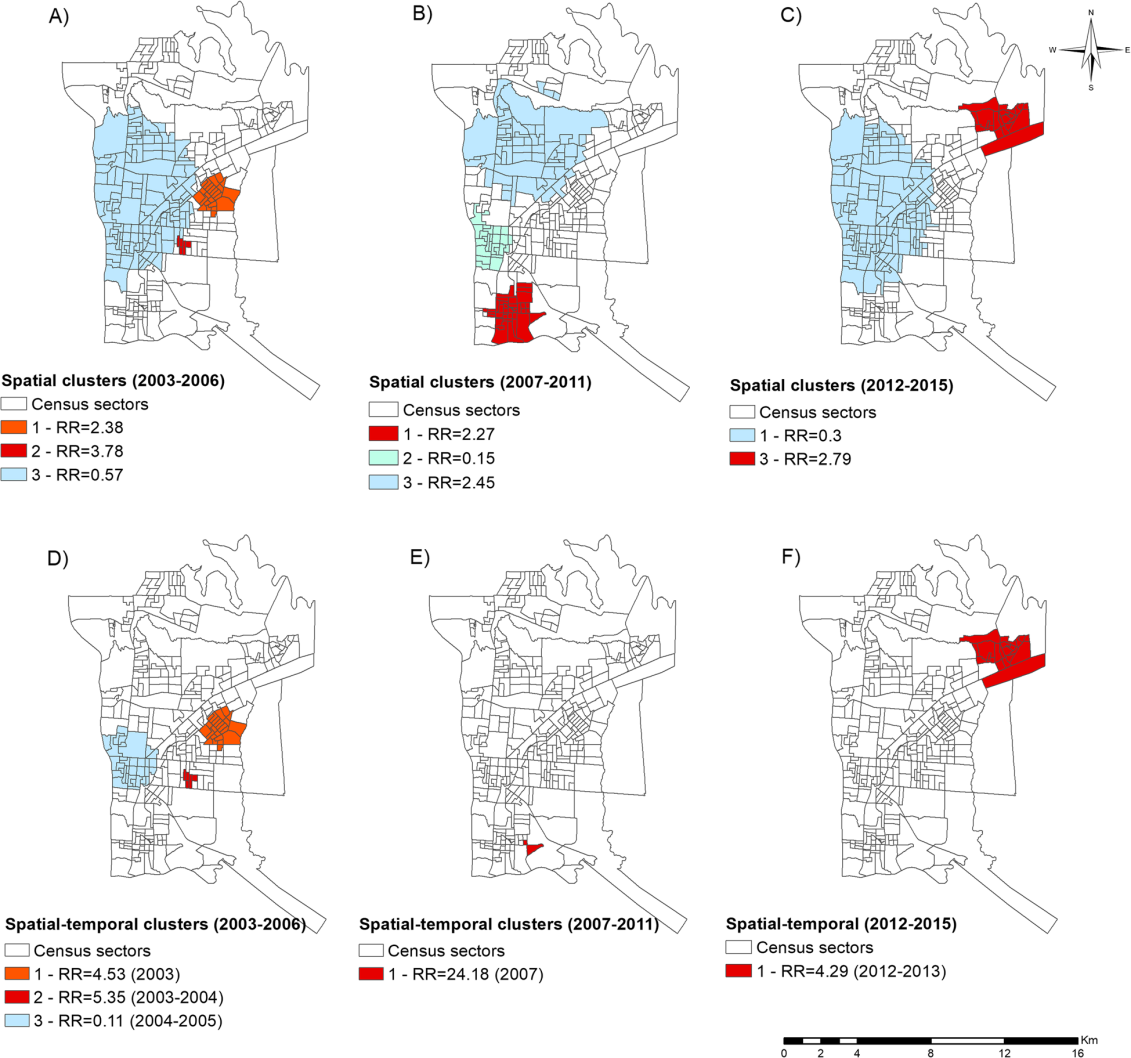
Dynamics of leprosy in three distinct periods in the triple-border region of Foz do Iguaçu, Paraná, Brazil. **a** Spatial clusters for the period 2003–2006, **b** spatial clusters for the period 2007–2011, **c** spatial clusters for the period 2012–2015, **d** spatial-temporal clusters for the period 2003–2006, **e** spatial-temporal clusters for the period 2007–2011, **f** spatial-temporal clusters for the period 2012–2015. Source: Created by the main author

Fig. 3 presents the dynamics of the chain of leprosy in the border region, where it is possible to compare the three distinct periods; the differences between the periods are described in more detail in Table 1. It is important to analyze each map in Fig. 3 and compare them with their statistics in Table 1.

**Table 1:** Main characteristics of the risk areas identified and evidence of the leprosy dynamics in the triple border area of Argentina, Paraguay and Brazil

Type of analysis and period	Cluster	Number of sectors	Number of cases	Population	Detection rate (100,000 inhabitants)	RR	*P*-value	95%CI
Spatial 2003–2006	1	24	55	18,426	74.6	2.38	0.001	1.78–3.17
	2	5	21	4212	124.6	3.78	0.003	2.43–5.87
	3	127	85	91,387	23.3	0.57	0.013	0.44–0.73
Spatial 2007–2011	1	37	55	25,781	42.7	2.30	0.002	1.70–3.09
	2	25	3	18,108	3.3	0.15	0.009	0.04–0.46
	3	63	27	50,569	10.7	0.45	0.036	0.30–0.67
Spatial 2012–2015	1	136	23	96,734	5.9	0.30	0.001	0.19–0.47
	2	21	27	18,818	35.9	2.79	0.043	1.83–4.23
Spatial-temporal 2003–2006	1 (2003^a^)	24	27	18,426	146.6	4.53	0.001	3.05–6.71
	2 (2003–2004^a^)	5	15	4212	177.9	5.35	0.008	3.19–8.97
	3 (2004–2005^a^)	33	2	24,364	4.1	0.11	0.046	0.02–0.44
Spatial-temporal 2007–2011	1 (2007^a^)	2	6	1207	497.4	24.18	0.038	10.78–54.21
Spatial-temporal 2012–2015	1 (2012–2013^a^)	21	21	18,818	55.8	4.29	0.0040	2.70–6.81

^a^Period of occurrence of the spatial-temporal cluster

Source: Created by the main author

According to Fig. 3a, specifically for the period from 2003 to 2006, two spatial risk clusters were identified, both located in the Eastern District of the study setting. Cluster 3, in the Western District and part of the Northern District, was considered low-risk.

Figure 3b, for the period from 2007 to 2011, also presents three spatial clusters: two, in the Western and Northern District, were considered low-risk for leprosy and one cluster in the Southern District was identified as high-risk. In the period from 2012 to 2015 (Fig. 3c), one low-risk spatial cluster (cluster 1) was formed in the Northern and Western District and one relatively high-risk spatial cluster (cluster 2) in the Northeast District.

Regarding the spatial-temporal scan statistic for the period from 2003 to 2006 period (Fig. 3d and Table 1), two high-risk clusters were found in the Eastern District of the municipality, with cluster 1 occurring specifically in 2003 and cluster 2 occurring in the period from 2003 to 2004. Cluster 3 was considered low-risk and located in the Western District, specifically in the period from 2004 to 2005. From 2007 to 2011 (Fig. 3e), one high-risk spatial-temporal cluster was formed in the Southern District, specifically in 2007. For the period from 2012 to 2015 (Fig. 3f), one high-risk spatial-temporal cluster was formed in the Northeast District between 2012 and 2013.

**Fig. 4:**
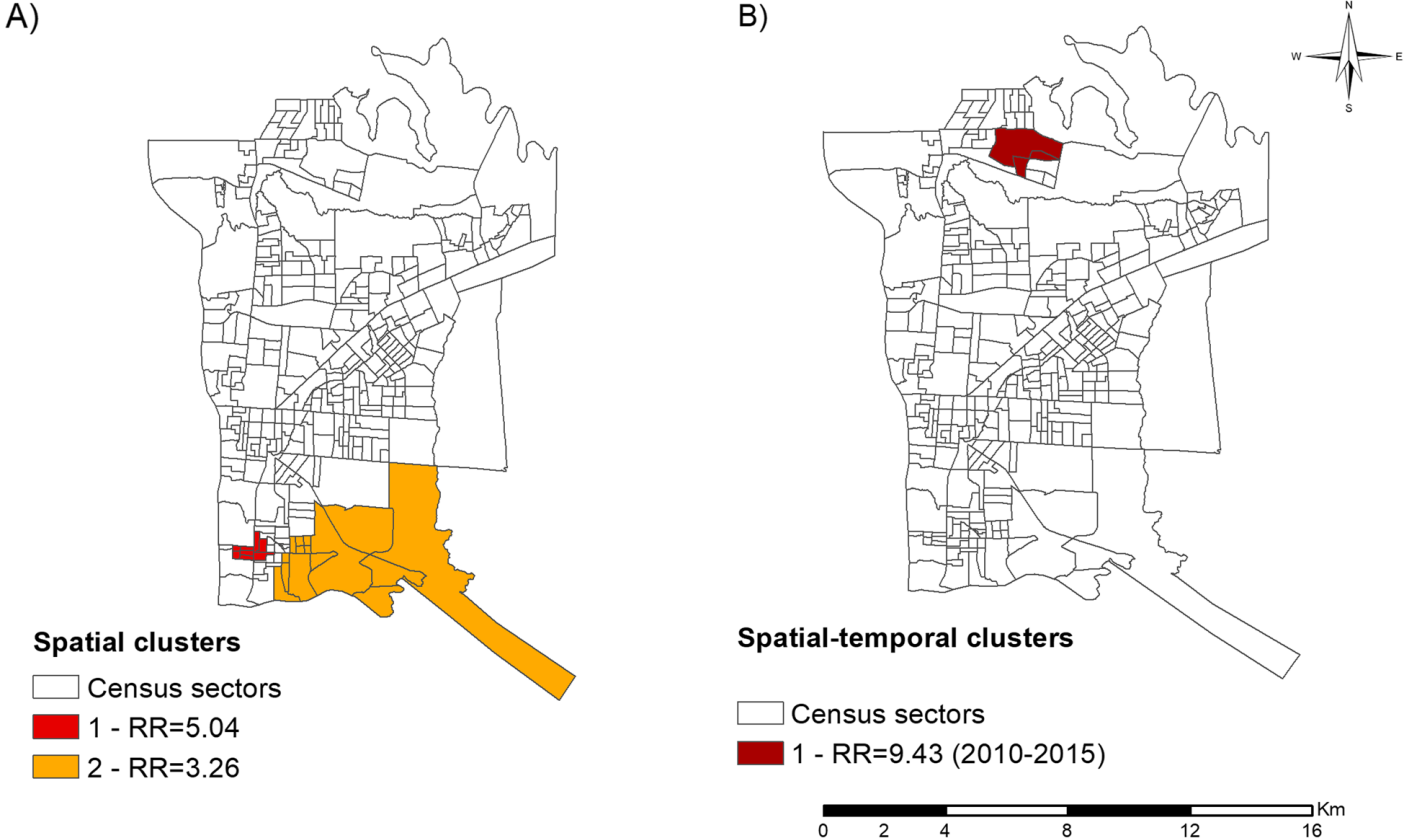
Areas of risk for leprosy disabilities in the Brazil, Paraguay and Argentina border region (2003–2015). **a** Spatial analysis, **b** Spatial-temporal analysis. Source: Created by the main author

The results in sequence (Fig. 4) highlight the critical areas for physical disabilities. Figure 4a shows the high-risk spatial clusters for G1D and G2D, located in the Southern and Western Health Districts (Cluster 1 - RR = 5.04, 95%CI = 3.05–8.31, *p* = 0.001; Cluster 2 - RR = 3.26, 95%CI = 2.11–5.01, *p* = 0.002). Figure 4b shows a spatial-temporal risk cluster (RR = 9.43, 95%CI = 4.82–18.44, *p* = 0.016) in the period from 2010 to 2015, located in the Northeastern District of the municipality.

### Kernel density estimation

**Fig. 5:**
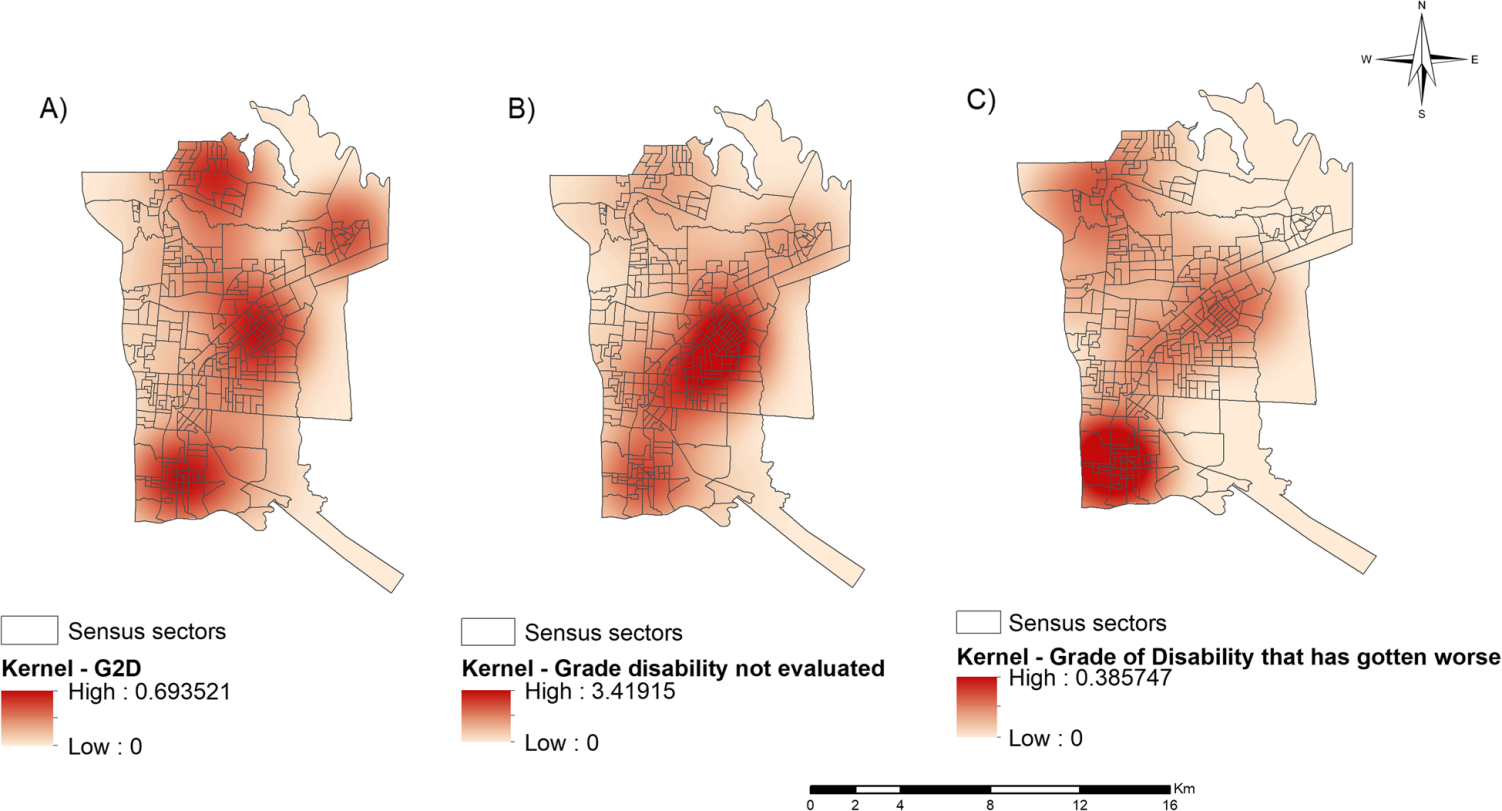
Spatial analysis of the leprosy disabilities in the Paraguay, Argentina and Brazil border region (2003–2015). **a** Density of leprosy cases with grade 2 disability at diagnosis, **b** Density of cases of leprosy in which the grade of disability was not reported/investigated at the time of diagnosis, **c** Density of leprosy cases in which there was worsening of the disability condition. Source: Created by the main author

In addition, considering the number of cases with G2D (*n* = 54) at the time of diagnosis, a spatial analysis of this condition was chosen using the Kernel Density Estimation. Figure 5 (a-c) presents the maps resulting from this analysis.

Figure 5a shows that the hotspots were concentrated in the Northern, Eastern and Southern Health Districts, presenting 0.693 cases per km^2^.

An analysis was also performed for those areas in which the disabilities were not investigated at the time of diagnosis or in which this information was missing when the data were obtained. Figure 5b shows the critical areas for this indicator. Clearly, this situation was more concentrated in the Eastern Health District, presenting an epicentre with approximately 3.419 cases per km^2^.

The areas with cases in which the disability worsened were also investigated. Figure 5c shows the region where the grade of disability was developing, that is, the grade of disability increased between the time of diagnosis and the time of cure, with a higher density of cases (0.385 cases per km^2^) located in the Southern Health District of the municipality.

## Discussion

The aims of the study were to analyse the dynamics of leprosy, determining whether the leprosy risk areas changed over time, showing those areas where disabilities were more frequent and identifying the regions in which the patients experienced worsening of the grade of disability in a city located on the Brazil, Paraguay and Argentina triple border. The results of the study show changes in the pattern of risk areas for the occurrence of leprosy in the periods investigated, where areas are in constant transformation, known as the territory process [42]. Another relevant finding that may be related to late diagnosis refers to the physical disabilities present among the investigated cases. In some risk areas, patients show worsened physical disability after diagnostic confirmation, which raises the hypothesis of inadequate clinical monitoring and reinforces the need to structure leprosy control services in a qualified manner, considering the integral nature of all of the diagnosed cases.

The information related to the monitoring of signs, symptoms and possible complications and the systematic registration of the clinical evaluation and/or information regarding the presence of a reaction episode should be recorded in the medical records in its entirety. Furthermore, this highlights the lack of information in the notification form regarding the impairment of neural function (grade of disability), according to the recommendations of the Handbook on Prevention of Disabilities of the Ministry of Health in Brazil [43].

By means of the spatial scan statistic, two risk clusters for the occurrence of leprosy located in the Southern and Eastern Districts of the municipality were identified in the period from 2003 to 2015, while the spatial-temporal scan indicated a risk cluster in the Eastern District in the period from 2011 to 2015. Using the Getis-Ord Gi* technique, it was possible to observe hotspots, mostly in the Southern District. Regarding the region identified with the Gi* technique, it should be noted that it is known to include a commercial and financial pole, with foreign relations with Argentina (border region), a region with much migration, poverty and a high population density [44].

These results are in agreement with other studies that relate high rates of leprosy detection to low socioeconomic and environmental conditions [8, 45–47]. In addition, other factors need to be considered, such as low educational levels, a lack of access to health services, low income with which to purchase medications that are supplementary to the treatment, the overcrowding of housing, housing without basic sanitation, and a lack of access to adequate food [46, 48–50].

In the spatial-temporal analysis (Fig. 2b), it was possible to identify a leprosy risk cluster in the period from 2003 to 2007, located in the Eastern Health District. This region is characterised as being one of the first large subdivisions of the municipality, inhabited by former employees of the Itaipu Binational Power Plant and of the civil construction, which is responsible for the urban development of the municipality [44]. However, when the analyses were performed for the three different periods (Fig. 3), it was possible to identify other spatial and spatial-temporal clusters, confirming changes over the years. The period from 2003 to 2007 was highlighted as having a mean detection rate of new cases of leprosy of 36.63 per 100,000 inhabitants [28].

This period and the high rates of detection of new cases coincide with high detection rates in the state of Paraná. According to the Department of Health Surveillance of Paraná, in one year (2007) there was an increase in units that diagnose and treat leprosy in the state of 81.9%. For 2006, 1400 new cases of leprosy were diagnosed in Paraná; of these diagnosed cases, 1.92% were in patients under 15 years of age, 8.45% had severe physical disabilities at the time of diagnosis, and 63.78% presented advanced forms of disease [51].

In 2010, the process of decentralization of the programmes for controlling and combating tuberculosis and leprosy to PHC was started, initially in the Family Health Teams and gradually for other health units, in order to facilitate access of the population to diagnosis and treatment, making them available close to their homes [52]. Prior to this period, the leprosy actions were centralised in only the Medical Specialty Centre, which made it difficult for most of the population to have access to this service, and caused fear due to the stigma involved with this disease [53]. The results show the impact of the decentralisation process over the years, as changes of the clusters could be monitored. The potential of this process to improve access to populations is highlighted. In 2015, the Municipal Centre of support for tuberculosis and leprosy was created in the city of Foz do Iguaçu; however, the PHUs continued to treat the cases [54]. The main objective of the Support Centre is to reduce the mortality, morbidity and dissemination of disease and to promote early diagnosis, acting in conjunction with Primary Care and other municipal services (public or private) [55].

Areas considered to be low-risk and coldspot areas were mainly identified in the Western District and part of the Northern District. Regarding the grades of disability 1 (G1D) and 2 (G2D), through the spatial scan statistic it was possible to identify two clusters located in the Southern and Western District of the municipality; also, in the spatial-temporal analysis, a cluster was observed in the Northern District. The Kernel Density Estimation demonstrated higher G2D case density in the Northern, Southern, and Eastern Districts. It should be highlighted that the Kernel confirmed density for G2D in the Northern District, even though it presented a low-risk cluster.

The low-risk clusters for leprosy in the three analyses were located in the Western Health District and part of the Northern District. These regions are characterised as being composed of businesses, hotels, universities and administrative public bodies, with the population basically constituted by workers and former workers of the Itaipu Hydroelectric Power Plant, the tourism sector, banking network and informal workers, as well as covering the border region between Brazil and Paraguay. A zero-risk cluster in the Northern District was also identified through the spatial-temporal analysis for the period from 2012 to 2015; that is, during this period, there were no new cases of leprosy. The absence of cases in this period is worrying, as it may indicate faults in the diagnosis by the PHC, that is, it was not prepared to make the diagnosis, making the region silent for new cases of leprosy.

As of 2012, there was a decrease in the rate of detection of new cases of leprosy in the municipality, with a mean rate of 15.13 cases per 100,000 inhabitants [52]; however, this is still considered high by the Ministry of Health. This fall may be related to the process of decentralisation of the program for the combat and control of leprosy, which began its implementation in 2010, suggesting that PHUs need training in leprosy management, with early diagnosis being extremely important to prevent the development of physical disabilities.

Regarding G1D and G2D, through the spatial scan statistic, this study revealed two risk areas in the Southern and Western Districts. A spatial-temporal cluster was also identified in the period from 2010 to 2015 in the Northern District. Through the Kernel Density Estimation, only the G2D cases presented regions with higher density in the census sectors of the Southern, Eastern and Northern Health Districts.

In notes and/or bulletins from the municipality, a decline in leprosy rates has been observed in recent years; however, the number of cases with a diagnosis of Grade 2 disability is increasing, which may indicate a delay in diagnosis [52]. An issue beyond cases with disability is the low examination of Disability Grade. When analysing Fig. 5c, it can be seen that there was a higher density of cases not reporting disability at the time of diagnosis in the Eastern District, which may indicate the inability of health teams to perform a diagnosis.

In Fig. 5d, it is clear that the density of cases with evolving disability was higher in the Southern District and the density of cases with a stationary degree of disability was higher in the Eastern District; in these regions, it is possible that the health services are not effective with regard to treating physical disabilities, corroborating the studies of Lana, Carvalho and Davi [56] in the Vale do Jequitinhonha, Monteiro et al. [57] in Tocantins and Ribeiro, and Lana [58] in Diamantina-MG. It is essential that disability prevention actions occur in parallel with the measures of disease control.

One of the WHO’s goals is the reduction of new cases of leprosy with grade 2 disability [7]. Deformity caused by leprosy is a major public health problem, as it affects the economically active population and is often irreversible. Early diagnosis and immediate treatment may help to prevent the development or worsening of disabilities in leprosy cases [4]. Continuous education should be a strategy implemented by the management, aiming for qualification of the healthcare providers, thus contributing to the identification of cases, considering that while there are regions with more cases diagnosed, other regions remain silent, perhaps due to the active search in strategic points of the areas and communities still not being prioritised [4].

A previous study carried out by Assis et al. [7] in Foz do Iguaçu showed that households with a monthly per capita monthly income of more than 1 minimum wage have a lower risk of illness and people of brown race are at higher risk of becoming sick with leprosy. The findings confirm the relationship between social determinants and leprosy.

It should be emphasised that social and health inequalities are phenomena with great power in the chain of disease progression, as well as an obstacle for some populations to access health systems and services. The literature is vast in terms of active search activities and their impact on breaking the chain of the transmission of leprosy; however, the care models have prioritised acute conditions (or acute chronic conditions), which may be related to the data found concerning the number of patients detected with G1D and G2D.

Another issue that should be mentioned is the number of leprosy cases that, even after being diagnosed, presented worsening in their degree of disability, reinforcing the idea of a technical-care model focused on the acute condition and not investing in measures of supported self-care, education and health promotion. Family empowerment may lead to the reversal of this situation; however, there must be a unique therapeutic project constructed for each patient that seeks the health services, which lack time, skills and support of the management.

In terms of advancing knowledge, this study provides support for the comprehension of the disease in this region, indicating priority areas for health actions. Among the limitations is the use of secondary data obtained from SINAN, which may present instability and incomplete data due to the failure to complete notifiable disease forms.

## Conclusion

In conclusion, leprosy is still a problem in the region and although the detection rates are decreasing, the findings indicate that this has not reached the stage of control. The spatial analyses performed showed areas of high-risk for the disease, which may indicate the active circulation of *Micobacterium leprae*. In addition, the occurrence of G2D cases in certain areas indicates late diagnosis, perhaps due to the inability of services to identify cases and a failure to prioritise the active search for symptomatic patients in the community. Another worrying condition is low-risk areas where leprosy may be silent. Surveillance, prevention and control actions, as well as the training of healthcare providers to diagnose cases, in both high and low-risk areas, are important if there is a clear intention to end leprosy and overcome the Brazilian image as being responsible for leprosy in America.

## Data Availability

The data were obtained from the Health Surveillance Department of the Health Department of Foz do Iguaçu, Paraná, Brazil. Data were provided for the survey but are not publicly available; data can be made available upon request to the Department of Health Surveillance. Data from the 2010 Demographic Census were collected through the website of the Brazilian Institute of Geography and Statistics (IBGE), referring to the census sectors.

## Abbreviations

CAAE: Certificate of Ethical Appraisal
ECU: Emergency Care Units
FDR: False Discovery Rate
G1D: Grade 1 disability
G2D: Grade 2 disability
HDI: Human Development Index
IBGE: Brazilian Institute of Geography and Statistics
ISA: Incremental Spatial Autocorrelation
PHC: Primary Health Care
PHU: Primary Health Units
RR: Relative Risk
SINAN: Notifiable Disease Information System
SUS: Sistema Universal de Saúde: Brazilian National Health System
WHO: World Health Organisation
